# The essential role of physician as advocate: how and why we pass it on

**Published:** 2017-06-30

**Authors:** LeeAnne M. Luft

**Affiliations:** 1 University of British Columbia, British Columbia, Canada; 2 Division of Infectious Diseases, Department of Medicine, Kelowna General Hospital, British Columbia, Canada

## Abstract

There is consensus amongst regulatory and certifying associations that the role of physician as advocate is a fundamental competency for Canadian physicians. Understanding what advocacy is and looks like in daily practice is integral to achieving this competency. Identifying barriers and exploring how we as physicians acquire the skills of advocacy are discussed. The current state of advocacy in medical education is reviewed as the starting point for exploring how best to foster the skills of physician as advocate.

“The good physician treats the disease; the great physician treats the patient who has the disease.”

-William Osler

## Advocacy - what is it?

As physicians, we often find ourselves at the crossroads of a unique and sometimes intimate knowledge of patient needs, intersecting with the ability to leverage influence to change health care system delivery, social barriers, and even impact political policy. It is incumbent on physicians to be very familiar with the concept of seeing patient needs beyond a biomedical model and incorporating social factors of health into patient care, which forms the foundation of being an advocate. These factors may include education, income and employment, social support and housing, as well as early childhood experiences.^1^ To what degree any individual patient is impacted by these factors is a complex interplay of familial, cultural, economic, and political forces, which means there are multiple dimensions for possible intervention. In recent years, a compelling case has been made for advocacy as part of professionalism.^2^,^3^ A proposed definition of physician advocacy should integrate both individual and societal facets of advocacy:

*Action by a physician to promote those social, economic, educational, and political changes that ameliorate the suffering and threats to human health and well-being that he or she identifies through his or her professional work and expertise.*^4^

Moving beyond professionalism expectations, the concept of physician responsibility as it relates to social responsibility is discussed in detail elsewhere, but at its most basic level, must begin with overall competency in the practice of medicine, as well as meeting duties and agreed upon standards as part of the role (e.g., professional and licensing bodies’ mandated advocacy activities).^5^ On an individual basis, the precursors of advocacy must first involve establishing a patient-provider relationship wherein the needs of the patient are understood and heard, followed by taking additional steps to ensure a patient receives the services they need to recover and thrive in society. It is essential to have the humility to recognize many physicians come from a background of relative privilege,^6^,^7^ and thus may not be able to directly relate to how a person without social privilege experiences illness or need. Being educated professionals, physicians ought to be reflective, self-aware, and cautious in assuming they know or understand the socially disadvantaged patient’s priorities. Once a provider-patient relationship has enabled the physician to adequately understand the needs and priorities, the core of advocacy is undertaking the necessary action(s) to bring about positive change for the patient or group of patients. Common advocacy actions at the level of the individual patient may include ensuring basic needs such as housing, nutrition and social supports as well as referrals for addiction programs are in place for the patient being discharged with no fixed address. On a larger scale, consideration must be given to the physician’s duty to work towards eliminating societal causes of health inequity on a population level. This can include educational programs highlighting a need or an unfounded social stigma, petitioning the public and private sector for direct health care resources and to help address societal inequities, lobbying government for financial and legislative reform, and many other activities elaborated upon elsewhere.^2^,^8^

The breadth of advocacy encompasses multiple levels; descriptive definitions may not do justice to all types of advocacy occurring in day-to-day practice.^8^ Patient-level activities are focused on an individual patient in a clinical setting with an immediate medical or social health need. Practice-level interventions include addressing societal and social inequities and quality improvement surveillance wherein change is implemented to impact the health of a population of patients. Community and system-level activities include activism with the goal of creating lasting change to a system or policy, and knowledge exchange where ideas are created, disseminated, or shared.^9^

Tension may arise between advocating for one’s individual patient and the unintended consequences to others in the system. If, as physician advocates, we fail to recognize system inequities and to petition for societal change, efforts made at the level of individual patient will necessarily be unending and at a possible cost of unintended negative consequences to others in the system.^10^ For instance, a clinician may go “above and beyond” in meeting a need for their individual patient, such as securing long term care placement, expediting surgical services, preferential mental health or addictions program enrollment, but if the service itself is in inappropriately short supply, this will come at a cost to others (e.g., other patients on a waitlist are impacted). Though advocating for the needs of the individual is appropriate and within the scope of practice, on a larger scale, physicians must also try to impact social determinants of health and the expansion of care and resources lest what is done for one patient comes at a cost to another. Acknowledging that resources will always be finite, effective advocates nevertheless ought to attempt to help as many patients as is feasible.

## Is physician advocacy important?

Numerous Canadian associations and committees have validated the integral role of physician as advocate, including the Canadian Medical Association,^11^ the College of Family Physicians of Canada (CFPC),^12^ and the Royal College of Physicians and Surgeons of Canada (RCPSC).^13^ Both the CFPC and the RCPSC list advocacy skills as fundamental competencies for Canadian physicians, described as responding to individual patient’s health needs by advocating within and beyond the clinical environment, and also to the needs of communities or populations for system-level change in a socially accountable manner.^13^ The RCPSC CanMEDS Physician Competency Framework further delineates the ways in which physicians are accountable to society:

*[…] recognize their duty to contribute to efforts to improve the health… not limited to mitigating illness or trauma, but also disease prevention, health promotion, and health protection. Improving health also includes promoting health equity, whereby individuals and populations reach their full health potential without being disadvantaged…*^13^

Challenges remain in how best to integrate these competencies into existing medical curricula, but there is increasing agreement that the skills of advocacy are essential and need to be addressed.^14^

If there is widespread agreement that physicians are required to be well-equipped advocates, it will be imperative to identify individuals with specific attitudes and strengths for medical school admittance.^15^ These are expanded upon by Hubinette et al., and include such qualities as altruism, passion and perseverance, a desire to be socially responsible, and a teachable attitude.^16^ Following enrollment, prioritizing foundational skills of advocacy must begin at the level of the undergraduate medical student. This was recognized in a 2001 Health Canada report describing the unique role for Canadian medical schools:

*The optimal preparation of future practitioners to respond to population needs; a leadership role in advocacy for the services and resources needed for optimal patient care; leadership in social accountability and the inclusion of the concept of social accountability in the accreditation process of medical schools.*^17^

## What are the barriers to physician advocacy?

An important first barrier to having engaged physician advocates is the selection criteria of medical schools traditionally placing heavy emphasis on biomedical knowledge and predictors of achievement as a medical expert. If our medical communities and training institutions place a high value on advocacy, pre-medical school experiences that highlight awareness of social justice issues and a desire to intervene will be increasingly considered.^7^ The paucity of evidence indicating physicians currently engage in civic matters for their patients is troubling. As described elsewhere, physicians are statistically less involved than other professions or the general public on civic matters as simple as voting.^4^,^18^ Several factors likely come into play to account for this discrepancy between what physician groups endorse as their assigned role, and what they actually do. Earnest et al. posits that medical training largely removes physicians from the community setting during a long, arduous, academically demanding formative period.^4^ Financial and time pressures in practice may make it difficult for practicing physicians to take on anything more than the pressing clinical problem at hand.

Although Canadian certifying bodies and regulatory associations have placed increased emphasis on advocacy skills, the described skill set has shifted from advocacy by the medical profession as a whole, to an expectation placed on each individual physician, and also from policy-level interventions to pursuing individual patient issues, as well as community and population needs by individual physicians.^19^ With the increased individual expectation placed on physicians, there has been a lack of clear description of the activities and behaviours the physician is expected to perform. This can contribute to physicians feeling overwhelmed by how and where to begin with such a daunting task. Fear of being ostracized and straying from guideline- and evidence-based medicine may also impact the willingness of physicians to be strong advocates. As discussed below, a framework of “collective” advocacy within a team of health care providers provides an effective model from which to practice and could reduce some of the onus placed on the individual.^20^,^21^ Expectations from institutions, licensing bodies, and remuneration systems may need to be adjusted to better support effective advocacy activities both by individuals, and especially within the context of a collective team.

## How do we enable physician advocacy in medical education?

It seems clear that the first step towards teaching junior colleagues how to advocate is to model the activity ourselves and thereby act in a mentorship role. Being explicit with learners about ongoing advocacy actions interwoven into clinical care, as well as identifying physician colleagues who exemplify the skills of advocacy can help clarify the skills desired. Personal experience often predisposes an individual to participate in advocacy, and can be further augmented by formal training and mentorship.^15^,^22^ As we acquire more objective studies on social determinants of health and their outcomes, this can increasingly be woven into medical school curricula as a core knowledge base. Going beyond knowledge, a qualitative study suggested that personal experience and morals may be more predictive of engagement in advocacy activities than professional codes of conduct.^23^

Traditionally, advocacy was something observed in predecessor physicians who were notable role models as advocates, then subsequent physicians began to experience and hone similar behaviors as independent practitioners. In more recent years, there is an increasing emphasis on service learning: engaging undergraduate medical students in community-based, hands-on projects to understand and become involved with social and societal health needs.^24^–^28^ This will require an awareness of community resources available and how best to form collaborations with organizations. It remains to be seen how these types of curricula will impact future practice patterns and attitudes, or the impact (whether beneficial or possibly harmful) on the communities of patients targeted by various projects.^29^ In 2001, the RCPSC noted there was a lack of an “operational” curriculum framework or any parameters for assessing competency in advocacy,^30^ which led to the CanMEDS competency roles from the RCPSC.^31^

As we strive to enable physician advocates, traditional conceptualizations of physicians working independently have been challenged and enriched by descriptions of the collective nature of advocacy.^19^–^21^ In a recent study, ten physicians, identified by their colleagues as being effective health advocates, described the collective nature of advocacy. Specifically, these accomplished physician advocates identified the need for inter-professional teams (i.e., social workers, nurses, occupational therapists, physiotherapists, other physicians) and networking to obtain resources and support, best served by collective problem-solving wherein multiple different perspectives are taken into consideration.^20^ Within a team-based approach, the individual physician has a well-defined and thus manageable role. If team-based advocacy is indeed more effective than individual advocacy, then it is imperative surround oneself with likeminded and effective advocators as well.

## What is the current state of advocacy in medical education?

The consensus by leading medical educators that early (i.e., undergraduate level), universally required, hands-on, community-based curricular interventions for learning advocacy are essential has spawned numerous programs that vary in their timing, frequency, and intensity across the undergraduate curriculum.^32^,^34^ A thorough review of curricular interventions aimed at training effective physician advocates is summarized elsewhere.^16^ Given that most of these curricula are relatively new, there are few evaluative studies looking at outcomes in training medical students to be authentic advocators, although qualitative reports on various initiatives seems overall positive.^35^ One such study which compared a community advocacy program with no formal advocacy curriculum for first or second year medical students found that although attitudes towards advocacy were similar between the two groups, those that participated in enhanced training had greater self-reported knowledge of community health needs, and higher scores in skill-related surveys reflective of the hands-on nature of advocacy training.^24^ Assessment of a community service-learning initiative at UBC study showed positive outcomes, as reported by participants, in terms of acquiring new skills and techniques which could be applied to patient education and management as well as addressing health disparities.^28^

Post-graduate needs assessment surveys on nonclinical core competencies have also highlighted a deficiency in training, familiarity, and skills acquisition related to advocacy. Of concern, a qualitative Canadian review suggested that the Advocate role was considered the least relevant of the CanMEDS competencies by both educators and learners, further compounding the problem.^36^ A nationwide Canadian questionnaire to obstetrics and gynecology trainees reflected that most trainees (95%) felt health advocacy was important to address during training, but only 36% reported their training needs were addressed. Awareness of community resources and groups was reported in 27%, and only 20% had participated in community advocacy programs during their residency.^37^ Given the awareness and interest expressed in the advocacy role, it seems an opportune time to formally integrate advocacy activities and skills into all postgraduate curricula.

Still at the post-graduate level, results of pilot programs specifically designed to address advocacy skills have been described, and evaluated. An American orthopedics residency program designed a series of lectures and journal clubs on specific advocacy-related topics and concepts. Specific topics addressed included geriatric advocacy and Medicare, orthopedics-industry relationships, orthopedic state-specific advocacy, and under- and uninsured patients.^38^ Prior to curriculum implementation, 76% of participants (n=21) indicated they had never received any specific advocacy training, with 100% indicating they felt they should receive such training. Statistically significant (p<0.05) shifts in perceptions were seen in multiple realms as reported in pre- and post-curriculum questionnaires; whether advocacy was important to their education, whether health policy was important to patients, the relevance of underinsured and uninsured advocacy, and state-level advocacy needs as being relevant.

## How can we foster the skills of physician advocate?

With the assumption that there is consensus that advocacy is an essential skill for physicians and as such should be thoughtfully and specifically taught throughout medical training, the task becomes how to build an educational framework. We must begin by selecting future colleagues that have an interest in, and hopefully a passion for, the role of physician as advocate. Both trainees and “accomplished” physician advocates must constantly and humbly return to the person or group we are advocating with to ensure our priorities align and that we are indeed working alongside. This will require the ability to establish excellent therapeutic relationships built on trust, listening, and understanding. The ability to liaise with community organizations that do not necessarily have biomedical outcomes as their primary goal ought to be encouraged and modelled early during the formative training of future doctors. Hubinette et al. have described four categories essential to fostering advocacy skills including admissions, knowledge and skills curriculum, critical thinking and reflection, and experiential and workplace learning.^16^

As fledgling curricula are rolled out internationally, it will be exciting to follow in upcoming years which interventions best meet the needs of trainee physicians, and in turn, patients and our communities. The importance of practicing physicians as role models/teachers has perhaps been underutilized to date, at least in a formal curriculum setting. As trainees obtain knowledge in the importance of social determinants of health, learn within an expectation to participate in advocacy activities, and are aware how to access the resources in their community, there still may be a translational gap of putting theory into practice. The willingness of health care providers with expertise and experience in melding clinical practice with robust advocacy is tremendously valuable in the role of mentor.^39^ As such, educational institutions must engage strong physician advocates as clinical teachers, and place as high a value on the skill set as is currently given to medical expert-based biomedical knowledge or procedural techniques. This may require acknowledgement by educational institutions and provincial healthcare systems to protect the non-remunerable time required in addition to basic clinical service.

The interpersonal and collective nature of advocacy may not lend itself to rigid guideline-based practice expectations, but conversely, identifying measurable outcomes will be imperative. Some residency training programs have introduced targeted advocacy training in a similar two-week “block” style to traditional subspecialty rotations, which although important, may prove to be insufficient in terms of the desired-for skill set that should be interwoven into daily clinical interactions.^40^ Using the CanMEDS competency roles from the RCPSC, the boundaries between physician as advocate, communicator, and collaborator can become blurred when one observes physician advocacy champions functioning within a collective team. Physicians cannot neglect the holistic skill set needed to succeed. At some point, mandated curriculum will always have its limitations and there is a certain onus on the individual trainee physician to seek out and reflect upon individual patient needs and how it intersects with the social privilege of being a medical professional. This self-motivated maturity will always be difficult to objectify, but we can still set measurable outcomes. Defining success in advocacy is not straightforward and the input of accomplished physician mentors, community organizations, and indeed the patients with whom physicians advocate alongside, all will be important gauges of success.

The increasing interest in medical advocacy education seems an encouraging indicator that within the medical community there are gifted, motivated, and experienced physician advocates. Interestingly, one of the pressing needs now is for this group to “advocate for advocacy.” This will entail not only setting specific educational goals at the UME and PGME level, defining experiences in advocacy, providing structure at examination levels, integration into existing residency programs, but also recognition of the inherent value of advocacy and acknowledgement of the time it often requires.^41^

## Conclusion

Despite its diverse and broad definition, physicians often intuitively know when advocacy is required and that it is integrally important to the health of the individual and in caring for the greater community. The both tacit and official acknowledgement by multiple licensing bodies should serve to reaffirm what we, as medical professionals, already knew. Acquisition of advocacy skills is complex, longitudinal, inter-relational, and collaborative. The nature of the activity will necessarily mean it is harder to define, quantify, and assess than other traditional physician skills such as scholarship and technical medical expertise; hence, curricula are diverse and in evolution. Lastly, and perhaps most importantly, advocates must approach each patient as an individual and with a great deal of humility. Perception of need and how best to advocate is a lifelong challenge, which even the accomplished clinician advocate will find humbling and instructive.
